# In Loving Memory of Professor Joachim Messing, a Pioneer from Molecular Genetics to Plant Genomics

**DOI:** 10.1186/s12284-019-0343-5

**Published:** 2019-12-26

**Authors:** Yin Li, Jennifer Ayer, Paul Fourounjian, Jiaqiang Dong, Zhiyong Zhang, Chenxu Liu, Fan Feng

**Affiliations:** Waksman Institute of Microbiology, Rutgers, The State University of New Jersey, Piscataway, NJ USA

We deeply grieve that our beloved and respected mentor and colleague, Dr. Joachim (Jo) Messing, the member of Advisory Board of *Rice*, passed away at the age of 73. He was a University Distinguished Professor at Rutgers, The State University of New Jersey (Rutgers University hereafter), Director of the Waksman Institute of Microbiology, and the Selman A. Waksman Chair Professor of Molecular Genetics. Jo is survived by his wife Rita, son Simon and his wife Lisa, and three grandchildren (Daniel, Lukas and Henry). He is also survived by sister Angelika; a devoted staff; and many colleagues and friends.

Messing was born on Sep.10th 1946 in Duisburg, Germany. Jo grew up in a working-class family, amid the ruins and food insecurity that followed World War II. This childhood experience would foster his later ambitions to enhance food security and end world hunger. Messing received his Bachelor of Science and Master of Science in Pharmacy in 1968 and 1971, respectively. Following the suggestion of Nobel Laureate biochemist Feodor Lynen that “the future would be in understanding DNA,” he chose molecular biology for his doctorate study, working on the replication of DNA plasmids. Messing obtained his doctorate degree at the Ludwig-Maximillian University of Munich in 1975. After he trained as a postdoc at the Max Planck Institute of Biochemistry for 3 years (1975–1978), Messing decided to move to the United States, which he felt was very much supportive of young scientists. He first landed a research associate position at the University of California, Davis (UC Davis) in 1978. In 1980, Messing was recruited by the University of Minnesota-Twin Cities as an associate professor, and was soon promoted to a full professor in 1984. In 1985, Messing joined Rutgers University as a university professor of molecular biology and then became research director at the Waksman Institute of Microbiology. Since 1988, he had served enthusiastically as the director of the Waksman Institute until passing away unexpectedly on Sep 13th. 2019.

Messing made pioneering and foundational contributions to molecular genetics, genomics and evolutionary biology. Since 1974, he had continuously worked on the development of a molecular cloning system derived from the filamentous coliphage M13 and then host strains of *E.coli*. At UC Davis, he completed a full set of cloning toolkits (namely the M13mp/pUC/JM kit) implemented with a blue-white screen to facilitate clone selection, including plasmid strains, host vectors and universal primers. With these molecular tools in hand, Messing sought to perform multiple sequencing reactions on fragments of DNA in parallel, then allow computer software to connect these sequences by their overlapping regions into a long, intact DNA molecule. Messing named the technology “shotgun” DNA sequencing. As a proof-of-concept, the 8031-bp DNA of cauliflower mosaic virus was successfully sequenced in 1981 in collaboration with Bob Shepherd, a plant pathologist at UC Davis. Notably, the fact that Messing distributed his work for free, instead of patenting them, eventually fostered rapid innovation in sequencing from basic research to industry. The invention and applications of “shogun” DNA sequencing helped to crack the genetic codes of living organisms from humans to plants, revolutionizing medicine and agriculture. The “shotgun” sequencing approach conceptualized massively parallel genomic sequencing, a strategy that was then adopted for use by the Human Genome Project and still applies today in the next generation and third generation DNA sequencing technologies.

The landmark completion of the Human Genome Project heralded the beginning of a post-genomic era of biology. Since then, Messing made important contributions to the development of Rutgers biology research. He led the Plant Genome Initiative at Rutgers (PGIR), which actively participated in several international and national plant genome sequencing projects, such as The International Rice Genome Sequencing Project (IRGSP) and the sequencing of agronomically or ecologically important monocot species including maize, sorghum, *Brachypodium* and *Spirodela* (greater duckweed). Messing was one of the earliest genomic scientists to suggest a coordinated and comprehensive plant genome initiative (PGI) and the initiator of a maize genome project in the U.S. Messing, together with several U.S. scientists, Ben Burr, Dick McCombie, Robin Buell, and Rod Wing made major contributions from to the rice genome project (IRGSP), among which Messing contributed to the sequencing of several regions on chromosome 10, 11 and 12.

With the knowledge of these major crop genomes and comparative genomics in hand, Messing’s ambition of fighting hunger came into bloom. More recently, Messing’s lab focused on understanding maize seed development and improving nutritional, agronomic and bioenergy traits of crops, such as maize, sorghum and duckweed. He made important contributions to the cloning, identification and gene structure study of the major storage proteins in maize, zeins, and to the genetic modification of nutritional qualities through manipulation of zeins and their regulators.

Due to his many scientific achievements, Messing became the most frequently cited scientist in the 1980s. Jo won the 2013 Wolf Prize in Agriculture and 2014 Promega Biotechnology Award. He was a Fellow of the American Association for the Advancement of Science and the American Academy of Microbiology. Dr. Messing was also a member of both the U.S. and German National Academies of Sciences. He directed enormous energy into serving the scientific publications. He was the executive editor of the journal *GEN*E, editor of Current Opinion in Biotechnology and Methods, and a member of the Advisory Boards of BMC Plant Biology, Molecular Plant and Rice.

Messing’s professional excellence was matched by his generous and inspiring spirit as a colleague and friend. He was dedicated to making his work available freely to the scientific community without any restrictions, even before publication. Messing mentored and trained a great many scientists, whose achievements gave him pride. Today many of his lab alumni have become established professors and scientists in the US and countries around the world such as China, Mexico, Germany, Philippines, Switzerland, Romania and Kenya. Every day, his enthusiasm and passion for science created a truly positive and creative environment felt in his lab. Dr. Joachim Messing’s passing is a tremendous loss to the community. He will be remembered as an excellent scientist, inspiring mentor, great collaborator, loving husband, father and grandfather.



